# Development of a TaqMan Based Real-Time Fluorescent Quantitative PCR Assay for Detection of Porcine Cytomegalovirus in Semen

**DOI:** 10.1155/2020/5673145

**Published:** 2020-01-10

**Authors:** Rujing Chen, Qiuyong Chen, Xuemin Wu, Yongliang Che, Chenyan Wang, Longbai Wang, Shan Yan, Lunjiang Zhou

**Affiliations:** Institute of Animal Husbandry and Veterinary Medicine, Fujian Academy of Agriculture Sciences/Fujian Animal Disease Control Technology Development Center, Fuzhou 350013, China

## Abstract

This study described a TaqMan based real-time fluorescent quantitative PCR (qPCR) method to detect porcine cytomegalovirus (PCMV) infection, targeting the conserved region of the DNA polymerase (DPOL) gene. The standard curve showed a linear regression relationship with a coefficient of 0.999 and a slope of *y* = −3.249*x* + 38.958 corresponding to the amplification efficiency at 99.8%. The limit of the qPCR method was 51.9 copies/*μ*l. The established qPCR method showed excellent specificity, with no cross-reaction observed with common porcine pathogens. The coefficient of variation for intra-assay and interassay variability ranged up to 1.51% and 2.24%, respectively. PCMV positive signals can be found in semen using this qPCR method, which suggested that we should pay more attention to PCMV contamination in semen in order to eliminate PCMV infection in artificial insemination and xenotransplantation.

## 1. Introduction

Xenotransplantation was proposed to alleviate the current shortage in human donor organs available for allotransplantation [1, 2]. However, xenotransplantation may be associated with the transmission of porcine zoonotic microorganisms, including porcine endogenous retroviruses (PERVs), porcine cytomegalovirus (PCMV), porcine hepatitis E virus (HEV), and other porcine lymphotropic herpesviruses (PLHV, containing PLHV-1, PLHV-2, and PLHV-3) [3–5].

Porcine cytomegalovirus (PCMV), also called *Suid herpesvirus* 2 (SuHV2), which can cause acute to subacute disease characterized by fever, reduced general condition, anorexy, apathy, respiratory (i.e., sneezing, coughing, and dyspnoea), neurological signs, with the morbidity reaching nearly 100% and higher mortality to suckling piglets [6]. Previously research revealed that PCMV induces silent infection in adult pigs usually, but PCMV is an immunosuppressive pathogen that mainly inhibits the immune function of the macrophage and T cell lymphatic systems, which can lead to reproductive failure in pregnant sows [7]. These data indicate that it is essential to detect PCMV infection with rapidity, sensitivity, and specificity at an early stage in swine herds.

Several diagnostic methods, including virus isolation, polymerase chain reaction (PCR) methods [8–10], loop-mediated isothermal amplification assay (LAMP) [11], enzyme-linked immunosorbent assay (ELISA) [12–14], and Western-blot analysis [15] had been reported for detection of PCMV infection. However, these assays are either labor-intensive, less sensitive, require agarose gel analysis for the amplification products or had a risk of contamination, which may lead to false results. Real-time fluorescent quantitative PCR technology (qPCR) has become a powerful alternative platform for the detection and differentiation of pathogenic viruses [16–20]. In this study, a TaqMan based real-time fluorescent qPCR method, targeting the highly conserved DNA polymerase (DPOL) gene of PCMV, was developed for the rapid and reliable diagnosis of PCMV infection in porcine semen.

## 2. Materials and Methods

### 2.1. DPOL Gene Analysis

The virus (designated as strain PCMV-FJ01) was identified by us previously [21], and then the virus was isolated using the same strategy described by Gu et al. [6]. The complete DNA polymerase (DPOL) gene of PCMV was amplified by using the primers with overlapped three fragments (DPOL-1, DPOL-2, and DPOL-3, Table 1), which encompassed the complete DPOL gene of PCMV. The overlapped DPOL gene fragments were harvested with Gel Extraction Kit D2500 (Omega Bio-Tek, Guangzhou, China), then T-A cloned using pMD™18-T Vector Cloning Kit (Takara Biomedical Technology, Beijing, China). The positive recombinant plasmids were sequenced using the Sanger method by a commercial company (Sangon Biotech, Shanghai, China) in both directions. For each PCR product, three colonies were selected for Sanger sequencing (Sangon Biotech, Shanghai, China).

After Basic Local Alignment Search Tool (BLAST, https://blast.ncbi.nlm.nih.gov/Blast.cgi) search, the expected sequences were assembled with Lasergene package software (DNAStar, v7.1, Madison, WI, USA) [22] and then submitted to GenBank (https://www.ncbi.nlm.nih.gov/genbank/).

In this study, the DPOL gene of PCMV (FJ01 strain), other eight PCMV isolates (GenBank number: AF268039, AF268040, AF268041, AF268042, KF017583, HQ686080, HQ686080, and HQ113116), Human herpesvirus 6 (HHV-6) (U1102 strain, GenBank number X83413), Human herpesvirus 7 (HHV-7) (U1102 strain, GenBank number U43400), and Human betaherpesvirus 5 (HHV-5) (AD169 strain, GenBank number X17403) downloaded from the National Center for Biotechnology Information (NCBI, https://www.ncbi.nlm.nih.gov/nucleotide/) were used for further analysis. Phylogenetic trees based on the complete DPOL gene nucleotide sequences were constructed by using the neighbor-joining method using MEGA 6.05 [23]. Bootstrap analysis was performed with 1000 replications.

### 2.2. Primers and Probe Design

After a bioinformatics analysis of the DPOL gene of PCMV, specific primers-pair and a TaqMan probe were designed using Primer Premier Software version 5.0 (Premier Biosoft, Palo Alto, CA, USA). Detailed information regarding the primers (qPCMV-F and qPCMV-R) and probe (qPCMV-P) were shown in Table 1. The probe (qPCMV-P) was labeled with FAM and BHQ-1 at the 5′-terminal and 3′-terminal, respectively. The primers and probe were synthesized by a commercial company (Sangon Biotech, Shanghai, China).

### 2.3. Standard Plasmid Preparation

For real-time qPCR standard curve preparation, the linear recombinant plasmid (designated as T-DPOL-3, described in Section 2.1) was quantified using Thermo NanoDrop3300 spectrophotometer (Waltham, MA, U.S.A.) and copy number of T-DPOL-3 was calculated following the method described by Lee et al. [24]. The ten-fold dilution of the plasmid (from 5.19 × 10^8^ to 5.19 × 10^1^ copies/*μ*l) was stored at −80°C until use.

### 2.4. Real-Time qPCR Protocol

The optimization of conditions for real-time qPCR method was carried out in triplicates using TransStart Probe qPCR SuperMix (TransGen Biotech, Beijing, China) on Mastercycler ep realplex (Eppendorf, Hamburg, Germany), based on the manufacturer's instructions. Different concentrations of the primers and probe were prepared into reaction tubes to optimize the assay by evaluating the highest fluorescence and lowest threshold cycle (*C*_T_). The optimized reaction was carried out in a 20 *μ*l reaction system containing 10.0 *μ*l of supplied master mix, 0.4 *μ*l of each primer (qPCMV-F, qPCMV-R and qPCMV-P, 10 *μ*M each), 2 *μ*l templates DNA, and 6.8 *μ*l of Nuclease-free Water (TransGen Biotech, Beijing, China). The thermal profile for the real-time PCR was 94°C for 50s, followed by 40 cycles of 94°C for 5 sec, 60°C for 35 sec. The ten-fold dilution of plasmid from 5.19 × 10^8^ to 5.19 × 10^1^ copies/*μ*l was used as target DNA to generate the standard curve.

### 2.5. Sensitivity and Specificity Test

After optimizing the real-time qPCR reaction, 10-fold serial dilutions of plasmid DNA standards (ranged from 5.19 × 10^8^ to 5.19 × 10° copies/*μ*l) were used to confirm the detection limit (LOD).

Porcine circovirus 2 (PCV2), porcine parvovirus (PPV), porcine pseudorabies virus (PRV), porcine kobuvirus (PKV), porcine bocavirus (PBoV), *Escherichia coli* (*E. coli*), *Streptococcus suis* serotype 2 (*S. suis* 2, SS2), *Haemophilus parasuis* (Hps), and negative control (no template) were used to verify the specificity of the real-time qPCR method. The DNAs were extracted by TIANamp Virus DNA/RNA Kit DP315 (Tiangen Biotech, Beijing, China) for viruses (PCV2, PPV, PRV, PKV, PBoV) and Bacterial genome DNA Kit DP302 (Tiangen Biotech, Beijing, China) for Bacteria (*E. coli*, SS2, and Hps).

### 2.6. Reproducibility Analysis

The serial ten-fold dilutions of T-DPOL-3 (ranged from 5.19 × 10^8^ to 5.19 × 10^1^ copies/*μ*l) were used to evaluate the coefficient of variation (CV). For intra-assay variability, these T-DPOL-3 (ranged from 5.19 × 10^8^ to 5.19 × 10^1^ copies/*μ*l) were repeatedly tested three different times daily. For interassay variability, these T-DPOL-3 (ranged from 5.19 × 10^8^ to 5.19 × 10^1^ copies/*μ*l) were repeatedly tested at three different times weekly. The coefficients of variation (CVs) were calculated according to the formula of the geometric mean *C*_T_ values/standard deviation.

### 2.7. Detection of PCMV in Semen

Fifteen semen samples were collected from the PCMV positive swine herds, which was identified by us using indirect blocking ELISA for detecting antibodies against PCMV glycoprotein B (gB) gene descried by Liu et al. [14]. The semen samples were collected, transported on ice to the laboratory and stored at −20°C. Total DNA was isolated by using the Genorise Semen DNA Extraction Kit (GENORISE, Glen Mills, PA, USA), he conventional PCR method, amplifying a 413-bp fragment targeting the DPOL gene of PCMV [8], which was also used to test the clinical samples. The conventional PCR products were visualized by gel electrophoresis (1.0%). Then the PCR positive samples were T-A cloned and sequenced, and also the obtained sequences were compared with FJ01 strains to verify PCMV infection.

## 3. Results

### 3.1. DPOL Gene Analysis

The cloned DPOL gene of PCMV (FJ01strain) had the length of 3,021 bp. The DPOL gene sequences were submitted to the GenBank under the accession number MG696113. The nucleotide and deduced amino acid sequences of PCMV (FJ01strain) shared 98.9%–99.2% and 99.1%–99.7% with other eight PCMV isolates (GenBank number: AF268039, AF268040, AF268041, AF268042, KF017583, HQ686080, HQ686080, and HQ113116). Moreover, the nucleotide sequences of the PCMV-FJ01 DPOL gene shared 56.4%, 56.0, 51.0% with HHV-6 (strain U1102), HHV-7 (strain U1102 and HHV-5 (strain AD169), respectively.

**Figure 1:**
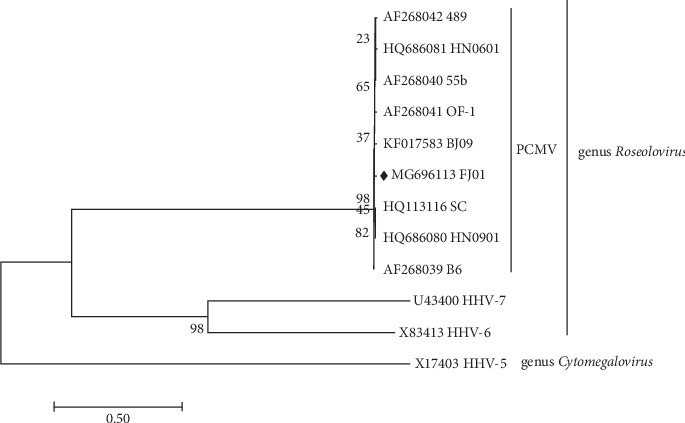
Phylogenetic tree based on the complete DPOL gene nucleotide sequences. The tree was generated by MEGA 6.05 software, using neighbor-joining method (bootstrap = 1000). The PCMV strain (FJ01) in this study was indicated with diamond (♦). Reference sequences obtained from GenBank are indicated by accession number and strain name. Genus was indicated on the right side of the tree.

Phylogenetic analysis based on the complete DPOL gene nucleotide sequences demonstrated that all the PCMV isolates share very close relationship, belonging to the same cluster together with HHV-6 and HHV-7 under the genus *Roseolovirus*, rather than HHV-5 (under the genus *Cytomegalovirus*). These data suggested that the PCMV can be classified as a member of the genus *Cytomegalovirus* under the subfamily *Betaherpesvirinae* using DPOL gene (Figure 1), which is in accordance with the phylogenetic tree based on the gB gene [25].

### 3.2. Sensitivity of Real-Time qPCR Method

**Figure 2:**
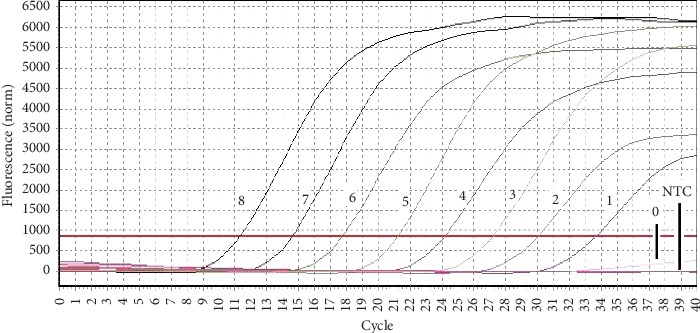
Sensitivity test of the real-time qPCR method. 8-0: PCMV plasmids with the concentration of 5.19 × 10^8^ to 5.19 × 10°copies/*μ*l; NTC: no template control.

**Figure 3:**
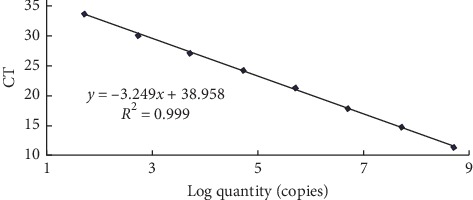
The standard curve of the real-time qPCR method.

The ten-fold dilutions of plasmid from 5.19 × 10^8^ to 5.19 × 10^1^ copies/*μ*l were used to generate the standard curve (Figure 2). The linear regression curve was plotted with the mean *C*_T_ (average of three) values on the *Y*-axis and logarithm values of the copy number of plasmids on the *X*-axis covering linear range. The curve showed linear regression relationship with a coefficient of determination (*R*_2_) of 0.999 and a slope of *y* = −3.249*x* + 38.958 corresponding to the amplification efficiency of 99.8% (Figure 3). As shown in Figure 2, the real-time qPCR method could detect up to 5.19 × 10^1^ copies/*μ*l per reaction indicating sensitivity of the assay.

### 3.3. Specificity of Real-Time qPCR Method

**Figure 4:**
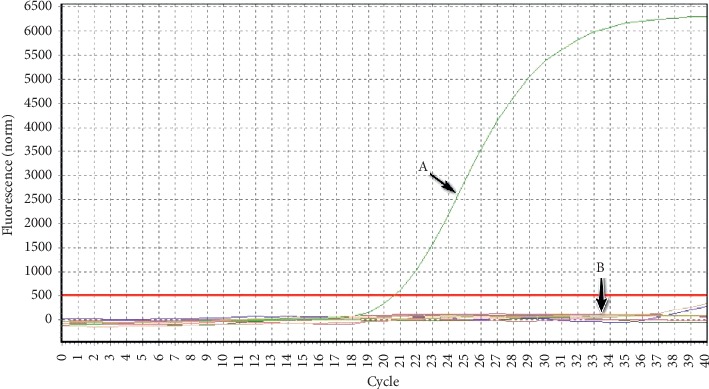
Specificity test of the real-time qPCR method. (a) Means the PCMV positive, (b) means the common porcine pathogens, such as Porcine circovirus 2 (PCV2), Porcine parvovirus (PPV), Porcine pseudorabies virus (PRV), Porcine kobuvirus (PKV), Porcine bocavirus (PBoV), *Escherichia coli* (*E. coli*), *Streptococcus suis* serotype 2 (*S. suis* 2, SS2), *Haemophilus parasuis* (Hps), and negative control.

As shown in Figure 4, strong fluorescent signal was obtained from reactions with PCMV, the negative control (no template) and other common porcine pathogens (PCV2, PPV, PRV, PKV, PBoV, *E. coli*, SS2, and Hps) were observed with no positive signal. These data indicated the real-time qPCR method with excellent specificity.

### 3.4. Reproducibility of Real-Time qPCR Method

As shown in Table 2, the CVs for intra-assay and interassay variability ranged from 0.57% to 1.51% and 0.65% to 2.24%, respectively, which were all less than 5%. These data indicated the capability of the assay to generate excellent reproducible results.

### 3.5. Detection of PCMV in Semen

As shown in Table 3, the positive detection rate for the real-time qPCR method and conventional PCR was 26.67% (4/15) and 13.33% (2/15), respectively. All PCMV positive samples in the conventional PCR assay were as well as positive in the qPCR assay. The conventional PCR positive samples were then cloned and sequenced to verify with PCMV infection. The cloned sequences showed nucleotide identities of 99.6% and 99.7% with FJ01 strain (GenBank Number MG696113).

## 4. Discussion

Currently, research on PCMV genomic diversity is lacking, because there was only one PCMV complete genome in the GenBank (BJ09 strain, GenBank number: KF017583) [6]. Glycoprotein B, an important transmembrane glycoprotein, which plays an important role when PCMV enters host cells, may cause the induction of humoral and cellular immune responses. The gB gene is highly conserved within the *Herpesviridae* family, and is widely used as a candidate gene in herpesvirus species identification and phylogenetic analysis [26–28]. Here, in this study, the nucleotide and amino acid sequences comparison demonstrated that DPOL gene sequences in different PCMV isolates are highly conserved, with no significant variation between DPOL nucleotide and amino acid sequences from different PCMV isolates. Phylogenetic tree based on DPOL gene sequences from different PCMV isolates showed closer relationship, which belong to the genus *Roseolovirus* rather than genus *Cytomegalovirus*. These data indicated porcine cytomegalovirus may be renamed as porcine roseolovirus.

Recently, twenty-seven viruses come from diverse families, some of which can cause disease in humans and were detected in human semen, which suggests that the viruses in semen are probably more widespread than currently appreciated [29]. Artificial insemination (AI), which is widely used assisted reproductive technologies in pigs, causing us to pay more attention to the quality of semen. Microbial contamination in semen may lead to be used as microorganisms, or pathogens causing specific diseases. It is well known that many viruses can be detected in porcine semen, such as African swine fever virus, Pseudorabies virus, Classical swine fever virus, Foot-and-mouth disease virus, and so on [30].

Though PCMV is not on the list of World Organisation for Animal Health (OIE, https://www.oie.int/), this implies that PCMV may be ignored, which may also cause extrem loss to the worldwide pig industry, especially for pigs as donor animals used for xenotransplantation. Viruses may also be transmitted to semen as a result of survival and replication within the accessory glands, which may also lead to disease through xenotransplantation. This is different from porcine HEV (genotype 3), a zoonotic potential factor, which can lead to a chronic infection in patients suffering from immunosuppressed syndrome [31]. In a previous preclinical experiment of transplanting kidneys into cynomolgus baboons and monkeys, the PCMV in kidneys led to early transplant failure. These data suggested that PCMV may be an indirect pathogen without infecting host cells. Though there was no evidence that PCMV can infect nonhuman primate and human cells, the transplantation failure may be due to cytokines produced in response to PCMV antigens [32].

In this study, we chose the DPOL gene as the target gene, which was used for primers and probe design to establish a TaqMan based real-time qPCR platform for the detection of PCMV, which was verified with no significant variation between different PCMV isolates. The developed TaqMan based real-time qPCR method showed high specificity (no cross-reactivity signals from other porcine pathogens were found), sensitivity (the limit of detection was 5.19 × 10^1^ copies/*μ*l), and reproducibility (intra-assay and interassay variability were less than 1.51% and 2.24%, respectively). The standard curve of the real-time qPCR method shared a linear regression relationship with a coefficient of 0.999 corresponding to the amplification efficiency of 99.8%. In the clinical investigation of semen collected from the PCMV sera-positive swine herds, the positive detection rate for the real-time qPCR method and conventional PCR was 26.67% (4/15) and 13.33% (2/15), respectively. All PCMV positive samples in conventional PCR assay were also positive in the real-time qPCR method. The conventional PCR positive samples were cloned and sequenced; these obtained sequences showed nucleotide identities of 99.6% and 99.7% with FJ01 strain. PCMV can be detected in the testis and epididymis [33], but to the best of our knowledge, shedding of virus in ejaculated semen has not been determined before. Porcine semen contaminated with PCMV poses a risk for breeding herds because AI technology can lead to fast spread with various sow population and also huge loss of disease-free status tissues and organs used for xenotransplantation [34]. Single layer centrifugation (SLC) method is easier to use, with time-saving compared with density gradient centrifugation, which can be used to remove bacteria and viruses, which was proved can remove more than 99% of PCV2 from semen [35]. But whether SLC technology also can reduce PCMV contamination in semen remains unclear, especially for the only qPCR positive (with lower copies number) semen samples. These obtained data by the established real-time qPCR method indicated that we should pay more attention to the herd management and prevent PCMV infection through semen.

## 5. Conclusion

In conclusion, the present TaqMan based real-time qPCR technique was established with high sensitivity, specificity, and reproducibility, which is convenient for screening large number of semen batches simultaneously for the presence of PCMV before they are used for artificial insemination and breeding programs. Further, this assay can be considered as a useful and practical tool for diagnosis and epidemiological investigation of PCMV.

## Figures and Tables

**Table 1:** The primers used in this study.

Primers	Abreaction	Sequences (5′ ⟶ 3′)	Position^*∗*^	Length (bp)
DPOL-1	DPOL-F1	ATGACATTCTTTAATCCATAT	3129–3149	1143
	DPOL-R1	TCCGACACCCAGCCTATACAAT	4250–4271	
DPOL-2	DPOL-F2	TGCCCACATCTATGAGTTTC	4109–4128	1152
	DPOL-R2	CTCTAGACGAAAGGACATTGT	5239–5259	
DPOL-3	DPOL-F3	TGACGGTGCATGTAAACAAGA	4933–4953	1222
	DPOL-R3	TTTTACATAACAATGACACT	6135–6154	

qPCR	qPCMV-F	GCGTAGAACTCGTTAGAA	5542–5559	188
	qPCMV-R	GCCAATTATGATGTCAATGATC	5709–5729	
	qPCMV-P	TCCGTTCCGTATCACTTCGTCG	5666–5687	—

^*∗*^The location sequence region of primers and probe was calculated based on GenBank number AF268039 (strain B6), the DPOL gene located at positions 3,129 to 6,152 of strain B6 (GenBank number AF268039).

**Table 2:** Reproducibility test of intra-assay and interassay for the real-time qPCR method.

Copies/*μ*l	Intra-assay			Interassay		
	*C*_T_	SD	CV (%)	*C*_T_	SD	CV (%)
5.19 × 10^8^	11.32	0.10	0.92	11.40	0.15	1.32
5.19 × 10^7^	14.65	0.12	0.80	14.70	0.13	0.91
5.19 × 10^6^	17.79	0.13	0.74	17.83	0.15	0.82
5.19 × 10^5^	21.26	0.12	**0.57**	21.32	0.15	0.68
5.19 × 10^4^	24.19	0.16	0.67	24.27	0.16	**0.65**
5.19 × 10^3^	27.26	0.16	0.58	27.35	0.21	0.77
5.19 × 10^2^	30.07	0.32	1.07	30.20	0.54	1.80
5.19 × 10^1^	33.64	0.51	**1.51**	34.08	0.76	**2.24**

**Table 3:** Detection porcine semen by conventional PCR and real-time qPCR.

Number	Positive samples		Copies/*μl*	
	Conventional PCR	qPCR	Both^#A^	only^#B^
15	2	4	7.35 × 10^4^ and 2.49 × 10^5^	6.18 × 10^2^ and 2.13 × 10^2^

^#A^Porcine semen were tested positive by conventional PCR and real-time qPCR. ^#B^Porcine semen were tested with conventional PCR negative and real-time qPCR positive.

## Data Availability

The data used to support the findings of this study are available from the corresponding author upon request.
